# More Energy and Less Fatigue: Implications for Successful Brain Aging

**DOI:** 10.1093/geroni/igaa057.2830

**Published:** 2020-12-16

**Authors:** Caterina Rosano, Nancy Glynn

## Abstract

Higher energy and lower fatigue are intuitively important to live independent, active lives. However, little is known about the relation between brain health, energy, and fatigue in older adults. From a neurobiological standpoint, energy and fatigue appear to rely on distinct, albeit overlapping brain networks, but most evidence is from patients with neurodegenerative conditions. These relations have not been fully examined in community older adults. In this symposium, we first present an overview of the neurobiology underlying fatigability and energy states. We will then present original unpublished data on brain health, fatigability, and energy from four well-established epidemiological studies of aging: Osteoporotic Fractures in Men Study (MrOS), Long Life Family Study (LLFS), Baltimore Longitudinal Study of Aging (BLSA), and the Health Aging Body Composition Study (Health ABC). Specifically, Ms. Allen will explore whether personality traits are related to perceived mental fatigability in MrOS. Using LLFS data, Ms. Gmelin will examine whether perceived physical fatigability is associated with global cognition, verbal fluency, memory and psychomotor speed. Dr. Schrack will share BLSA data showing cross-sectional and longitudinal associations between lower walking efficiency and reduced brain volumes. Dr. Tian will evaluate the neuroimaging signature of perceived energy levels in Health ABC. Taken together, our data indicate that higher energy and lower fatigability likely reflect overlapping but distinct aspects of brain health. The long-term effects of promoting energy and lowering fatigability on dementia should be further studied.

